# Risk Assessment of Foodborne Pathogens in Ready-to-Eat Poached Chicken in Shanghai, China

**DOI:** 10.3390/foods15162908

**Published:** 2026-08-19

**Authors:** Qiuping Tian, Hua Cai, Hao Zheng, Xiang Wang, Zhuosi Li, Yangtai Liu, Yue Ma

**Affiliations:** 1 School of Health Science and Engineering, University of Shanghai for Science and Technology, Shanghai 200093, China; 243452636@st.usst.edu.cn (Q.T.); 243452611@st.usst.edu.cn (H.Z.); xiang.wang@usst.edu.cn (X.W.); lizhuosi@usst.edu.cn (Z.L.); usstlyt@163.com (Y.L.); 2 Shanghai Municipal Center for Disease Control and Prevention, Shanghai 200336, China; caihua@scdc.sh.cn

**Keywords:** poached chicken, *Salmonella*, quantitative microbiological risk assessment, predictive microbiology, ready-to-eat

## Abstract

Shanghai-style poached chicken is a traditional ready-to-eat (RTE) dish in Shanghai. The locally monitored data revealed that the contamination rates of three major pathogens, including *Staphylococcus aureus*, *Listeria monocytogenes*, and *Salmonella*, were 8.33%, with *Salmonella* being the most frequently detected (4.76%), followed by *S. aureus* (2.38%) and *L. monocytogenes* (1.19%). A semi-quantitative risk assessment identified *Salmonella* as the key pathogen, with a high-risk score of 52. An established growth model for *Salmonella* isolates on poached chicken was utilized within a Monte Carlo simulation framework, combined with consumption data, to estimate ingestion levels per day across three typical consumption scenarios. Compared with dine-in consumption (Scenario 1), ambient storage (Scenario 2) and refrigeration (Scenario 3) increased the probability of infection by approximately 4-fold and 2-fold, respectively. Sensitivity analysis revealed the initial contamination level as the primary risk determinant, highlighting the importance of strict environmental hygiene in processing. Overall, this study provides a quantitative risk assessment of poached chicken in Shanghai and offers scientific evidence for targeted interventions along the food chain.

## 1. Introduction

Poached chicken, a traditional delicacy in Shanghai cuisine, is notably distinguished by its tender texture and unique flavor. The traditional preparation involves immersing a whole yellow-feathered chicken in water at approximately 95 °C for 15–20 min, which is shorter and at a lower temperature compared to other cooked meat products. Following rapid cooling in ice water, the chicken is sliced and consumed directly by dipping in sauce without further heating [1]. Consequently, while this mild thermal process preserves the sensory quality of the product, it might fail to inactivate foodborne pathogens completely [2]. In recent years, the growing consumption of poached chicken, often sold at delicatessen counters with varying hygiene standards, has raised public health concerns due to the potential pathogenic contamination during retail and household handling [3]. However, accurate risk assessments for Shanghai-style poached chicken remain scarce, leaving its pathogenic risk largely uncharacterized.

Previous risk assessments have indicated that *Salmonella*, *Staphylococcus aureus*, and *Listeria monocytogenes* are frequently high-risk pathogens in ready-to-eat (RTE) meat products [4,5,6]. For example, Altissimi et al. [7] identified *Salmonella* as a high-risk pathogen in dry fermented sausages in Italy. According to a risk assessment by Zhai et al. [8], three major pathogenic bacteria, including *Salmonella, Staphylococcus aureus* (*S. aureus*), and *Listeria monocytogenes* (*L. monocytogenes*), in RTE meat products in Shanghai collectively presented a moderate risk level. Shanghai-style poached chicken is hypothesized to share a similar pathogenic profile with general RTE meat products. Therefore, characterizing these three target pathogens is fundamental to a tailored risk assessment.

*Salmonella* is a genus of Gram-negative bacteria that commonly causes salmonellosis through the consumption of contaminated food. In China, *Salmonella* has been the predominant bacterial pathogen associated with foodborne outbreaks, accounting for over 90% of cases related to contaminated meat products [9]. Poultry and its derived products are widely recognized as predominant vehicles for foodborne *Salmonella* transmission [10,11]. The pathogenicity of *Salmonella* critically depends on its ability to invade host cells and the released toxins. Clinical symptoms of salmonellosis, such as acute diarrhea, fever, abdominal cramps, and vomiting, usually appear 6–72 h after ingestion and generally resolve spontaneously after 4–7 days. However, for susceptible subpopulations, *Salmonella* infection may lead to severe complications, such as bacteremia, dehydration, or even death [12,13]. Several dose-response relationships of *Salmonella* have been developed. The minimum infectious dose threshold for the general public is conventionally estimated at a relatively high level of 10^5^–10^6^ CFU [14]. In addition, a β-Poisson dose-response model ($P_{\mathrm{ill}}=1-(1+D/\beta {)}^{-\alpha }$, where α = 0.3126 and β = 2885 for the general population) is frequently adopted [15].

*S. aureus*, as a ubiquitous Gram-positive pathogen, poses a significant public health threat due to its potential to proliferate and secrete emetic enterotoxins in contaminated RTE foods [16,17]. *S. aureus* intoxication is characterized by a short period (30 min to 8 h) and the rapid onset of symptoms, including vomiting, diarrhea, and abdominal cramps, which typically subside within 24 h. When *S. aureus* concentrations reach approximately 10^5^–10^6^ CFU/g, the amount of preformed enterotoxins (approximately 0.1–1 μg) produced may be sufficient to cause staphylococcal intoxication [18,19,20].

*L. monocytogenes*, a ubiquitous Gram-positive foodborne pathogen, poses a critical public health concern due to its remarkable tolerance to diverse environmental stressors, such as refrigeration temperatures (0–4 °C), high salinity, and acidic conditions [21]. Despite its relatively low incidence rate, listeriosis is characterized by severe clinical outcomes and high mortality rates among vulnerable populations, including neonates, pregnant women, the elderly, and immunocompromised individuals [22,23]. In China, the case-fatality rate of listeriosis is as high as approximately 18%, with major clinical manifestations including severe invasive infections such as meningitis, septicemia, and fetal loss. The incubation period of *L. monocytogenes* is highly variable, ranging from a few days to several weeks. The dose-response relationship is characterized by a low infectious dose in susceptible hosts, with infection potentially occurring at levels below 10^3^–10^5^ CFU [24,25,26].

Risk Ranger, a well-established semi-quantitative risk assessment tool, is widely utilized to estimate the relative risk ratings and comparative rankings of foodborne pathogens [27,28]. For example, Hernandez-Jover et al. [29] determined that *E. coli* O157 and *Salmonella* spp. constituted the highest-risk pathogens in undercooked hamburgers, while *Listeria monocytogenes* emerged as the most critical pathogen within the RTE meat products. The resulting semi-quantitative risk assessment outputs provide a direct scientific foundation for prioritizing and implementing precise quantitative risk assessment (QMRA) of the predominant microbial hazards. QMRA is a structured framework that combines scientific evidence, data analysis, and mathematical modeling to quantify the probability of infection and disease associated with exposure to pathogens across different scenarios [30,31].

**Figure 1:**
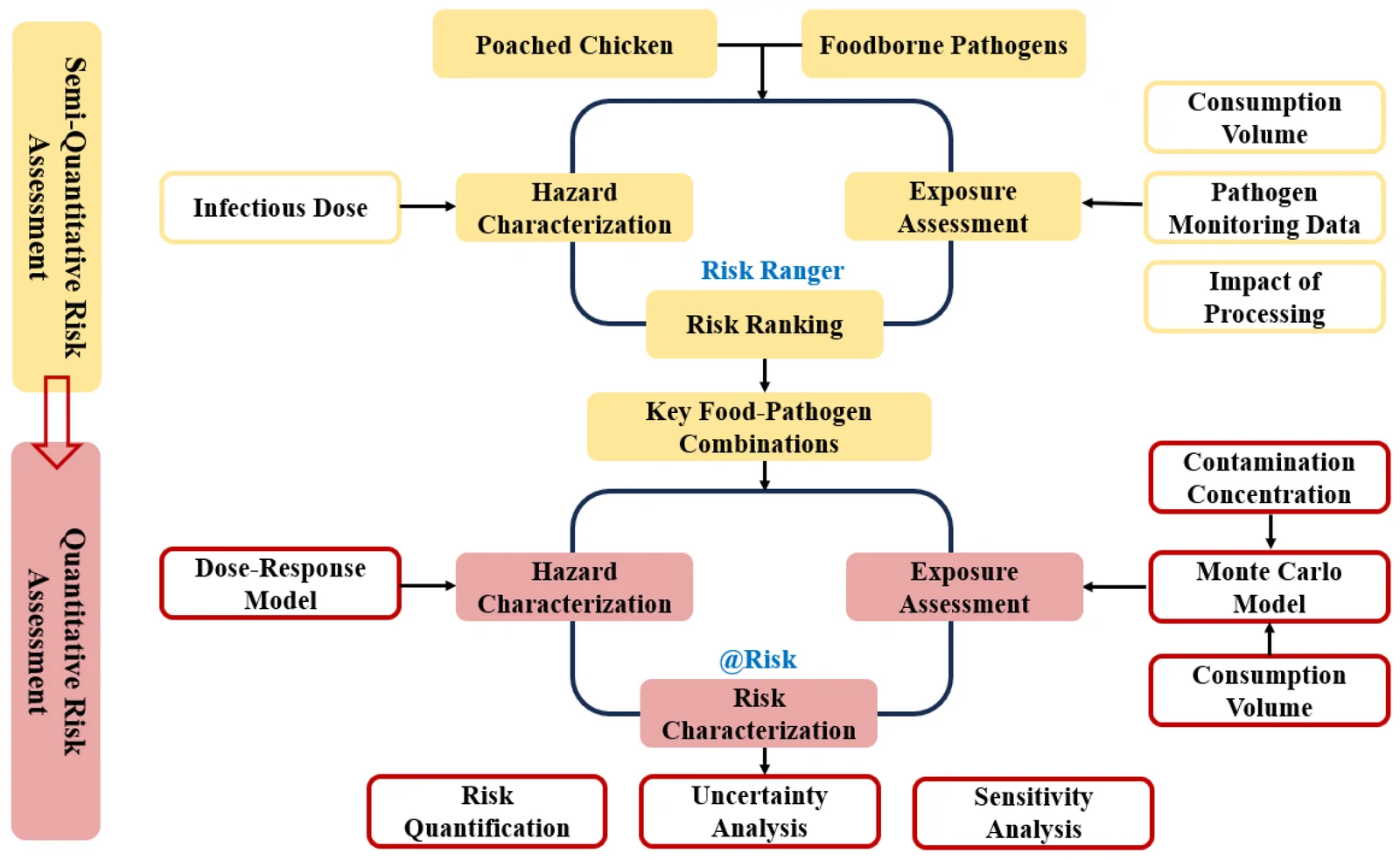
Risk assessment model in Shanghai-style poached chicken.

The overall methodological framework of this study is illustrated in Figure 1. To evaluate the risk of the poached chicken in Shanghai, this study quantified the prevalence and concentration of *Salmonella*, *S. aureus*, and *L. monocytogenes* in commercially available products. A semi-quantitative risk assessment was then employed to rank these pathogens and identify the primary hazard. With the key hazard identified, exposure assessment was conducted across different consumption scenarios, and the overall quantitative risk was estimated by integrating exposure outcomes with dose-response models. These findings offer a scientific basis for the risk management of poached chicken, a regionally characteristic RTE poultry product in Shanghai.

## 2. Materials and Methods

### 2.1. Sample Collection

In July 2025, a total of 84 poached chicken samples were collected from 13 administrative districts of Shanghai. Sampling sites were determined by the proportion of the district population. Retail outlets across districts (cooked food counters, general, and star-rated restaurants) were randomly selected for representative sampling. Without repeated sampling from the same source, each ~250 g sample from a unique establishment or batch was aseptically packed in a sterile stomacher bag, transported on ice, and processed immediately upon arrival.

### 2.2. Prevalence and Concentration Data

The prevalence and concentrations of *Salmonella*, *S. aureus*, and *L. monocytogenes* in commercially available Shanghai-style poached chicken were determined in accordance with the national standards GB 4789.4-2024 [32], GB 4789.10-2016 [33], and GB 4789.30-2025 [34], respectively. For qualitative analysis, 25 g of the poached chicken sample was homogenized with 225 mL of the corresponding enrichment broth in a sterile stomacher bag using a stomacher (BagMixer 400, Interscience, Saint-Nom-la-Bretèche, France) for 2 min. After pre-enrichment and selective enrichment according to the requirements of different pathogens, 100 μL of the diluted homogenate was streaked onto selective agars, including XLT4 agar (Qingdao Hope Bio-Technology Co., Ltd., Qingdao, China), Baird-Parker agar (Qingdao Hope Bio-Technology Co., Ltd., Qingdao, China), and Listeria chromogenic agar (Qingdao Hope Bio-Technology Co., Ltd., Qingdao, China), followed by incubation at the corresponding temperatures for 24 h. LOD = 10 CFU/mL. Suspected colonies were confirmed by biochemical tests or 16S rRNA sequencing.

For qualitatively negative samples, the Jarvis Equation (Equation (1)) was applied to estimate the representative mean concentration (D_m_); the estimated D_m_ was used as an anchor parameter for constructing a continuous cumulative distribution in @RISK (v8.2, Lumivero, Denver, CO, USA).

(1) $$D_{m}=-\frac{2.303}{V}log\frac{Z}{N}$$

where D_m_ is the mean concentration of pathogens below the LOD, *V* is the quantity of tested samples (g), *Z* is the number of negative samples, and *N* is the total number of tested samples.

For samples confirmed positive by qualitative testing, the corresponding reserved non-enriched portions were subsequently subjected to quantitative analysis. Specifically, the standard plate counting method was used to enumerate pathogens in qualitatively positive samples. 25 g of sample was homogenized with 225 mL of sterile phosphate-buffered saline to prepare a 1:10 homogenate, which was further serially diluted 10-fold. An aliquot of 0.3 mL from an appropriate dilution was spread-plated in triplicate onto the corresponding selective agar. After incubation under specified conditions, colonies were counted. The limit of quantification (LOQ) was 10 CFU/mL [35,36,37].

### 2.3. Semi-Quantitative Risk Assessment via Risk Ranger

Risk Ranger is a spreadsheet-based semi-quantitative risk assessment model developed in Australia and implemented within Microsoft Excel (Version 2021). By integrating a total of 11 input parameters (Q1–Q11) that encompass both qualitative indicators and quantitative inputs, the model generates a standardized public health risk score on a scale of 0 to 100 via built-in mathematical functions.

### 2.4. Growth Model of Salmonella

The *Salmonella* strain (ST32) used in this study was isolated from commercially available Shanghai-style poached chicken samples. The isolate was cultured in LB medium (Qingdao Hope Bio-Technology Co., Ltd., Qingdao, China) at 37 °C for 18 h. Then, 50 μL of bacterial suspension was inoculated onto 5 g of sterile chicken breast samples (heated at 95 °C for 15 min), reaching an initial loading concentration of approximately 4 log CFU/g. Inoculated samples were stored at six temperatures (15, 20, 25, 30, 35, and 40 °C), respectively. *Salmonella* counts were determined at predefined time points by plating serial dilutions on XLT4 agar (Qingdao Hope Bio-Technology Co., Ltd., Qingdao, China). Five independent biological replicates were performed for each temperature condition.

The growth profile of *Salmonella* was described using both the modified Baranyi model (Equation (2)) [36] and the Gompertz model (Equation (3)) [37].

(2) $$lgN_{t}=lgN_{max}+lg\left(\frac{e^{\mu_{max,ln}t}+e^{\mu_{max,ln}\lambda }-1}{e^{\mu_{max,ln}\lambda }-1+e^{\mu_{max,ln}\lambda }\cdot \frac{N_{max}}{N_{0}}}\right)$$

(3) $$lgN_{t}=lgN_{0}+lg\left(\frac{N_{max}}{N_{0}}\right)\times exp\left\{-exp\left[\frac{e\times \mu_{max,lg}}{lg(N_{max}/N_{0})}\times (\lambda -t)+1\right]\right\}\;$$

where $N_{t}$ is the bacterial concentration (CFU/g) at time t, $N_{0}$ is the initial bacterial concentration (CFU/g), *N*_max_ is the maximum bacterial concentration (CFU/g) (See Table S6 for details), λ is the lag phase duration (h), where *μ*_max_, _ln_ (h^−1^) in Equation (2) represents the natural-logarithm (ln) based maximum specific growth rate, and *μ*_max_,_lg_ (lg CFU/g/h) in Equation (3) represents the log_10_ (lg) based maximum growth rate. Because each model was parameterized independently from the primary growth data, no parameter conversion was required during the model fitting process.

The Huang square-root model (Equation (4)) [38] was used as the secondary model to describe the temperature dependence of the maximum specific growth rate.

(4) $$\sqrt{\mu_{max}}=a\times (T-T_{min})$$

where T is the temperature (°C), $T_{min}$ is the theoretical minimum temperature for growth, $\mu_{max}$ is the maximum specific growth rate (h^−1^), and a is the model parameter.

### 2.5. Exposure Assessment Model Overview

By integrating initial contamination levels with predictive microbiological models, the dynamic changes of *Salmonella* in Shanghai-style poached chicken from retail to consumption were successfully quantified and incorporated as inputs into the QMRA model. Based on on-site observations and customer interviews conducted during the sampling period in Shanghai, three exposure scenarios were established: (Scenario 1) Dine-in, (Scenario 2) takeaway followed by short-term ambient storage, and (Scenario 3) takeaway with prolonged refrigeration.

Informed by Shanghai summer meteorological data, resident commuting patterns, and household food-handling surveys [39,40], Pert distributions were used to characterize the temperature and time parameters at each stage, including retail storage, transport, and household storage. The specific parameter assignments were as follows: retail storage time (time1)~Pert(0, 4, 12) h and retail storage temperature (T_1)~Pert(0, 4, 10) °C; transport time (time2)~Pert(0, 1, 3) h and transport temperature (T_2)~Pert(4, 25, 37) °C; short-term ambient storage time (time3_1)~Uniform (0, 2) h and storage temperature (T3_1)~Pert(25, 32, 37) °C; and prolonged refrigerated storage time (time3_2)~Pert(2, 6, 24) h and temperature (T3_2)~Pert(0, 4, 25) °C. Here, temperature represents potential product temperatures in this scenario, bounded by an initial 25 °C before cooling and a minimum refrigeration level of 0 °C. The parameters of the exposure assessment model are presented and described in Table 1. The minimum, most likely, and maximum values of the PERT distributions were determined based on multiple data sources, including published literature, Shanghai summer meteorological observations, field investigations, consumer interviews, and expert knowledge regarding food handling practices. The corresponding sources and assumptions for each parameter are summarized in Table 1.

### 2.6. Consumption Data

To adopt a conservative approach to risk assessment, consumption data for Shanghai-style poached chicken were sourced from generic RTE meat product data derived from the 2023 Shanghai Resident Diet and Health Surveillance. Using a multi-stage stratified random sampling method, the survey collected 2900 valid samples, revealing an average RTE meat product consumption of 24.33 g/person/day.

### 2.7. Dose-Response Model

Several quantitative microbiological risk assessment studies have widely used the β-Poisson model for *Salmonella* dose-response assessment [41,42,43]. The general form of the β-Poisson model employed in this QMRA is represented by the following Equation (5):

(5) $$P(d)=1-{\left[1+\frac{d}{\beta }\right]}^{-\alpha }$$

where: P(d) represents the probability of infection; d is the intake dose obtained from exposure assessment; α and β are model parameters that describe the variability of host-pathogen interactions. In this risk assessment, the parameters (α = 0.3126, β = 2885) were adopted from Corbellini (2017) [15].

### 2.8. Risk Characterization

The designed QMRA model incorporates both variability and uncertainty in selected parameters. Input variables are characterized by probability distributions as described in Table 1. The risk assessment model was constructed using an Excel spreadsheet (Microsoft), and simulations were performed with @Risk software (version 5.5; Palisade, Newfield, NY, USA). Monte Carlo sampling with 100,000 iterations was conducted to propagate the variability and uncertainty of the model inputs. The final outputs of this QMRA model for the general population included the probability of infection per serving of Shanghai-style poached chicken (probability of infection from consuming one serving) under three typical consumption scenarios.

### 2.9. Sensitivity Analysis

A sensitivity analysis was conducted to examine the correlation between the variability of model input parameters and the probability of infection. Spearman rank correlation coefficients were calculated using a Monte Carlo simulation, and tornado graphs were generated to rank the importance of input distributions.

**Table 1:** Input variables, model equations, and probability distributions used for the quantitative exposure assessment of *Salmonella* in Shanghai-style poached chicken.

	@Risk Input Values	Variable Symbol	Unit	Probability Distribution or Formula	References
Initial contamination [Scenario 1, 2, 3]	Total *Salmonella* contamination rate in poached chicken	P_p_Shanghai	%	Beta (5, 81)	This study
	Total negative rate of *Salmonella* in poached chicken	P_n_Shanghai	%	1-P_p_Shanghai	This study
	Contamination level of *Salmonella*-positive samples	L_p_Shanghai	log CFU/g	Cumul(1.0, 4.53, {1.0, 4.53}, {0.75, 1})	This study
	Contamination level of *Salmonella*-negative samples	L_n_Shanghai	log CFU/g	Cumul(−6.42, 1.0, {−6.42, − 2.71, 1.0}, {0.01, 0.50, 0.99})	This study
	Initial pollution distribution	Y0_shanghai	log CFU/g	Discrete(L_p_Shanghai:L_n_Shanghai, P_p_Shanghai:P_n_Shanghai)	Calculate
Sales stage (Air conditioning on in summer) [Scenario 1, 2, 3]	Room temperature sales time	time1	h	Pert(0, 4, 12)	Field investigation + Measured
	Room temperature for sales	T_1	°C	Pert(0, 4, 10)	Field investigation + Measured
	Room temperature sales growth rate	μ_max 1	ln CFU/g/h	IF(T_1 > T_min, (a × (T_1 − T_min)^0.75 × (1 − EXP(b × (T_1 − T_max))))^2, 0)	Calculate
		A_tl	/	A(t) = time1 + (1/μ_max1) × LN(EXP(−μ_max1 × time1) + EXP(−h_0_) − EXP(−μ_max1 × time1 − h_0_))	Calculate
	Retail terminal pollution levels	Y_1	log CFU/g	Y0_Shanghai + μ_max1 × A_t − LN(1 + (EXP(μ_max1 × A_t) − 1)/EXP(Ymax − Y0_Shanghai))	Calculate
Transportation stage [Scenario 2, 3]	Transportation time from the restaurant to home	time2	h	Pert(0, 1, 3)	Customer survey
	Transportation temperature from restaurants to households	T_2	°C	Pert(4, 25, 37)	Meteorological data
	Transportation_growth rate	μ_max2	ln CFU/g/h	IF(T_2 > T_min, (a × (T_2 − T_min)^0.75 × (1 − EXP(b*(T_2 − T_max))))^2, 0)	Calculate
		A_t2	/	A(t) = time2 + (1/μ_max2) × LN(EXP(−μ_max2 × time2) + EXP(−h_0_) − EXP(−μ_max2 × time2 − h_0_))	Calculate
	Pollution level at the transportation terminal	Y_2	log CFU/g	Y(t) = Y_1 + μ_max2 × A_t2 − LN(1 + (EXP(μ_max2 × A_t2) − 1)/EXP(Ymax − Y_1))	Calculate
Short-term ambient storage stage [Scenario 2]	Storage time	time3_1	h	Uniform (0, 2)	Customer survey
	Storage temperature	T_3_1	°C	Pert(25, 32,37)	Cai et al. [44]
	Ambient storage growth rate	μ_max3_1	ln CFU/g/h	IF(T_3_1 > T_min, (a × (T_3_1 − T_min)^0.75 × (1 − EXP(b × (T_3_1 − T_max))))^2, 0)	Calculate
		A_t3_1	/	A(t) = time3_1 + (1/μ_max3_1) × LN(EXP(−μ_max3_1 × time3_1) + EXP(−h_0_) − EXP(−μ_max3_1 × time3_1 − h_0_))	Calculate
	Contamination level after household ambient storage	Y_3_1	log CFU/g	Y(t) = Y_2 + μ_max3_1 × A_t3 − LN(1 + (EXP(μ_max3_1 × A_t3) − 1)/EXP(Ymax − Y_2))	Calculate
Long-term refrigerated storage [Scenario 3]	Storage time	time3_2	h	Pert(2, 6,24)	Customer survey
	Storage temperature	T_3_2	°C	Pert(0, 4,25)	Cai et al. [44]
	Ambient storage growth rate	μ_max3_2	ln CFU/g/h	IF(T_3_2 > T_min, (a × (T_3_2 − T_min)^0.75 × (1 − EXP(b × (T_3_2 − T_max))))^2, 0)	Calculate
		A_t3_2	/	A(t) = time3_2 + (1/μ_max3_2) × LN(EXP(−μ_max3_2 × time3_2) + EXP(−h_0_) − EXP(−μ_max3_2 × time3_2 − h_0_))	Calculate
	Contamination level after household ambient storage	Y_3_2	log CFU/g	Y(t) = Y_2 + μ_max3_2 × A_t3_2 − LN(1 + (EXP(μ_max3_2 × A_t3_2) − 1)/EXP(Ymax − Y_2))	Calculate

## 3. Results

### 3.1. Prevalence and Concentration of Pathogens

As shown in Tables S1 and S2, a total of 84 Shanghai-style poached chicken samples were evaluated, yielding an overall pathogen prevalence of 8.33% (n = 7) for at least one target organism. Specifically, *Salmonella* was the most prevalent pathogen (4.76%, n = 4). *S. aureus* was identified in two positive samples (2.38%, n = 2). *L. monocytogenes* showed the lowest contamination rate (1.19%, n = 1).

Concentrations of pathogens in the positive samples were further quantified. As shown in Table S3. Notably, for *Salmonella* (n = 4), three samples were positive by the enrichment-based qualitative detection method but were below the LOQ during direct enumeration. For QMRA modeling purposes, these samples were assigned a reference concentration of 10 CFU/g (1.0 log CFU/g), corresponding to the LOQ of the quantitative method. The remaining quantifiable sample contained 4.53 log CFU/g.

### 3.2. Semi-Quantitative Risk Ranking

The detailed inputs for the semi-quantitative risk model are displayed in Table S4. Based on a preliminary characterization of pathogenic hazard profiles, the hazard severity (Q1) for the general population (Q2) was classified as “mild” for *S. aureus*, and “moderate” for both *L. monocytogenes* and *Salmonella* [18,45,46]. Due to the unavailability of specific data for Shanghai-style poached chicken, RTE meat consumption data (24.33 g/person/day) from the 2023 Shanghai Residents’ Diet and Health Surveillance was adopted, yielding a consumption frequency (Q3) of 4.11 days/100 g. The permanent population of Shanghai (Q5) was 24,802,600, and the proportion of consumers (Q4) was set at 75%, consistent with previous studies [47,48]. Given that the Shanghai-style poached chicken is consumed without reheating, the cooking step (Q7) was considered ineffective for pathogen inactivation. Re-contamination probability (Q8) was set at 1%, with no subsequent growth control measures (Q9), and preparation before consumption was assumed to not affect pathogen levels (Q11).

The risk assessment results are shown in Table 2. Both *S. aureus* (score 44) and *L. monocytogenes* (score 42) exhibited moderate risks. However, *Salmonella* received a high-risk score of 52, with an estimated daily probability of infection per person of 4.17 × 10^−7^ and 2830 annual cases in Shanghai. Consequently, *Salmonella* was identified as the primary risk hazard in Shanghai-style poached chicken, which was selected for the subsequent QMRA.

### 3.3. Predictive Growth Models of Salmonella

**Figure 2:**
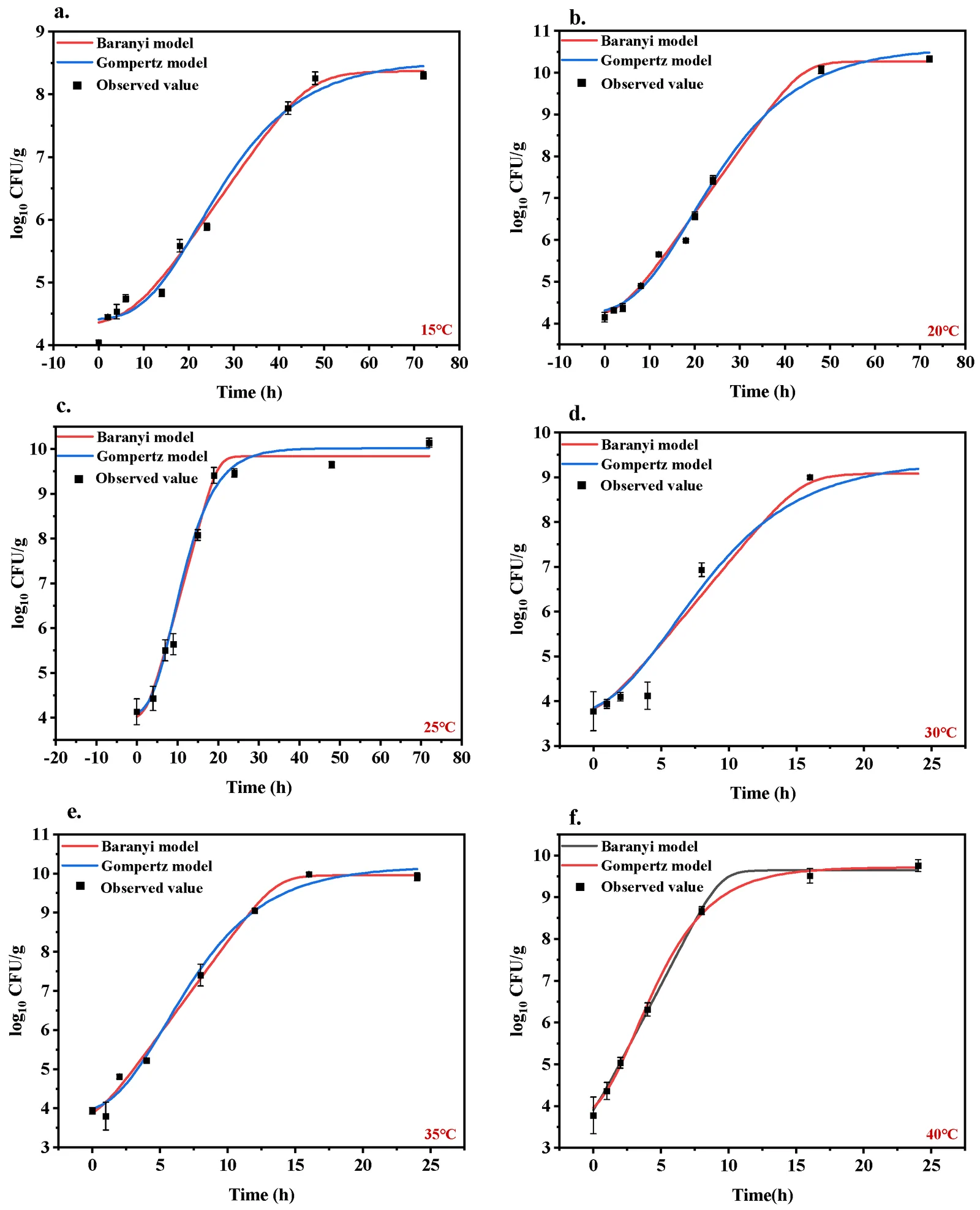
Growth curves of *Salmonella* in poached chicken samples at different temperatures ((**a**–**f**) correspond to 15, 20, 25, 30, 35, and 40 °C, respectively).

The development of a predictive growth model of *Salmonella* on Shanghai-style poached chicken is critical to support the subsequent exposure assessment. As shown in Figure 2, the dynamic growth curves of *Salmonella* in the poached chicken matrix always exhibited a characteristic sigmoidal profile across different temperatures. While both the Baranyi and Gompertz models demonstrated satisfactory fitting performance, the Baranyi model was selected due to its superior goodness-of-fit, as evidenced by lower RMSE and AIC values at most temperatures (Table S5). Accordingly, the kinetic parameters, including the maximum specific growth rate (*μ*_max_) and lag time (λ) across all investigated temperatures, were obtained based on the Baranyi model (Table 3).

**Figure 3:**
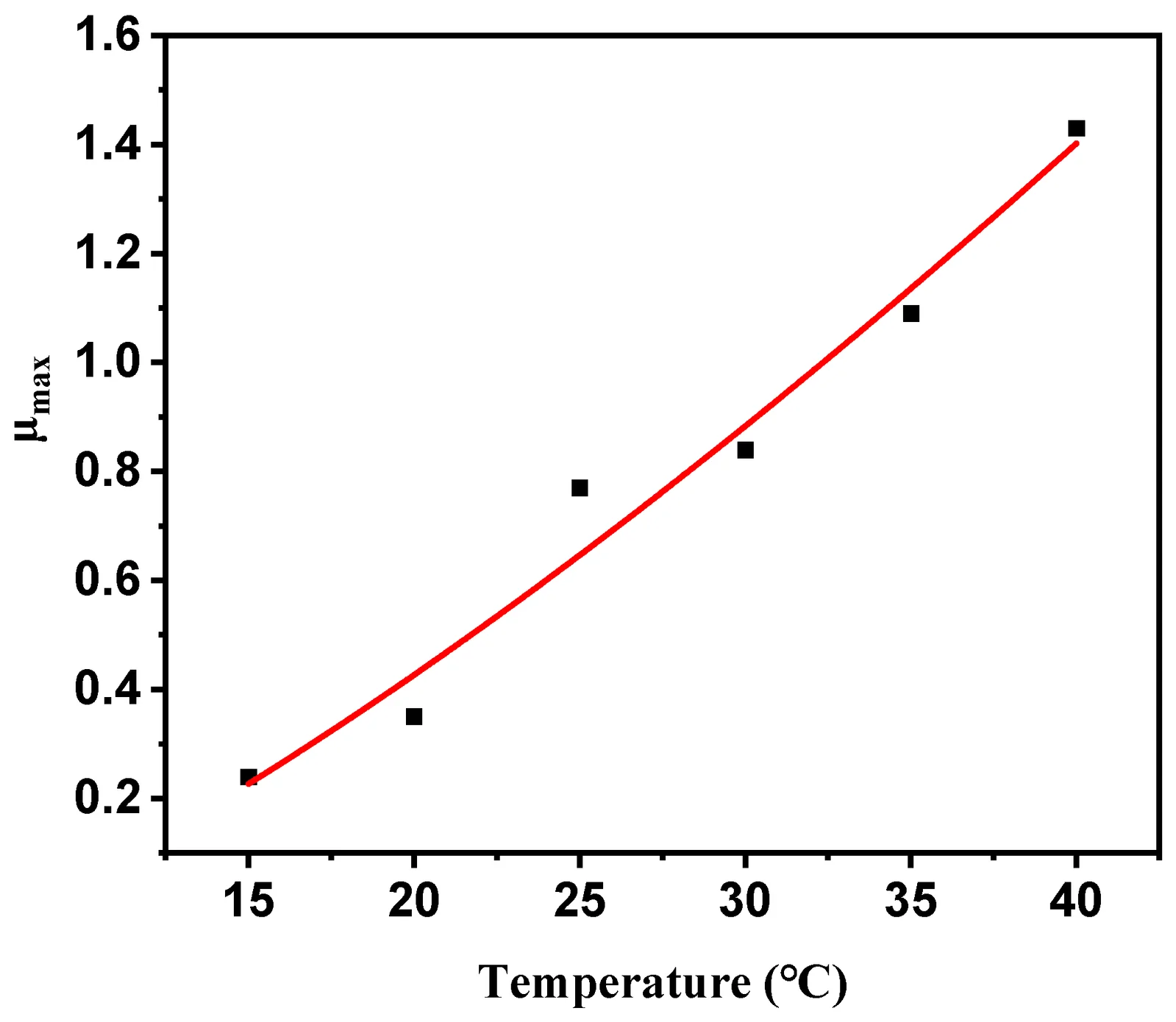
Secondary growth model of *Salmonella* in poached chicken using the Huang square-root model.

Moreover, the Huang square-root model was employed to develop a secondary growth model that characterizes the temperature-dependent kinetics of *Salmonella* in the poached chicken matrices. As shown in Figure 3, the model demonstrated a satisfactory fit to the experimental data with an RMSE of 0.0819. Consequently, the key secondary growth model parameters for *Salmonella* in poached chicken were determined, yielding a regression coefficient (a = 0.08 ± 0.01) and a theoretical minimum growth temperature (T_min_ = 3.71 ± 2.49 °C).

### 3.4. Exposure Assessment and Risk Estimates

**Figure 4:**
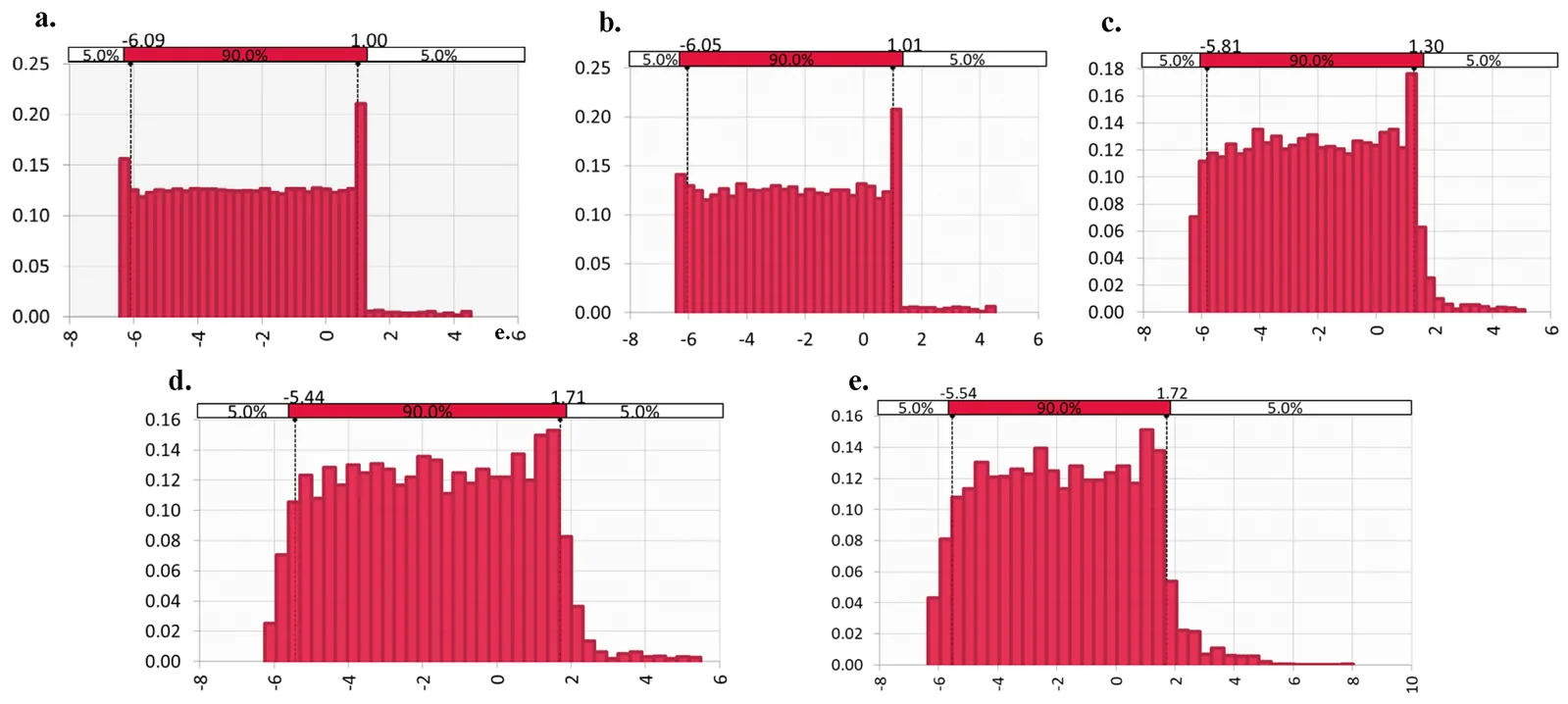
Distribution of *Salmonella* concentrations (log CFU/g) in poached chicken at each stage of three consumption scenarios. (**a**). Initial contamination; (**b**). After retail storage (Scenario 1); (**c**). After transportation; (**d**). Short-term ambient storage (Scenario 2); (**e**). Long-term refrigerated storage (Scenario 3).

Figure 4a depicts the initial *Salmonella* contamination profile, which yielded a mean level of −2.47 log CFU/g (5%: −6.090 log CFU/g~95%: 1.000 log CFU/g). In Scenario 1 (dine-in), after being held under specific conditions (T_1~Pert (0, 4, 10)°C; time1~Pert (0, 4, 12) h), the resulting *Salmonella* concentration distribution at the point of consumption is depicted in Figure 4b, exhibiting an increased mean contamination level of −2.43 log CFU/g (5%: −6.050 log CFU/g~95%: 1.010 log CFU/g). For Scenarios 2 (short-term ambient storage) and 3 (long-term refrigeration storage with initial cooling transient), the Shanghai-style poached chicken undergoes a transport stage from the retail to the domestic environment. Ambient temperatures and transit durations were modeled based on Shanghai’s meteorological records and average delivery times (T_2~Pert (4, 25, 37) °C; time2 ~Pert (0, 1, 3) h) [39,40]. Consequently, the resulting *Salmonella* concentration at the transport endpoint is presented in Figure 4c, where the mean contamination level increased to −2.15 log CFU/g (5%: −5.810 log CFU/g~95%: 1.300 log CFU/g). Building upon the contamination levels at the transport endpoint, *Salmonella* further proliferated during the subsequent home storage stages. Specifically, under Scenario 2 (short-term ambient storage with T_3_1~Pert (25, 32, 37) °C; time3_1~Uniform(0, 2) h) and Scenario 3 (long-term refrigeration storage with T_3_2~Pert (0, 4, 25) °C; time3_2~Pert(2, 6, 24) h), the average *Salmonella* concentration distributions further increased to −1.76 log CFU/g (5%: −5.440 log CFU/g~95%: 1.710 log CFU/g) and −1.04 log CFU/g (5%: −5.540 log CFU/g~95%: 1.720 log CFU/g), respectively (Figure 4d,e).

By integrating pre-consumption pathogen levels with consumption data using a dose-response model, the exposure estimates yielded daily *Salmonella* infection probabilities of 5.320 × 10^−6^, 2.234 × 10^−5^, and 1.168 × 10^−5^, respectively (Table 4).

### 3.5. Results of Sensitivity Analysis

**Figure 5:**
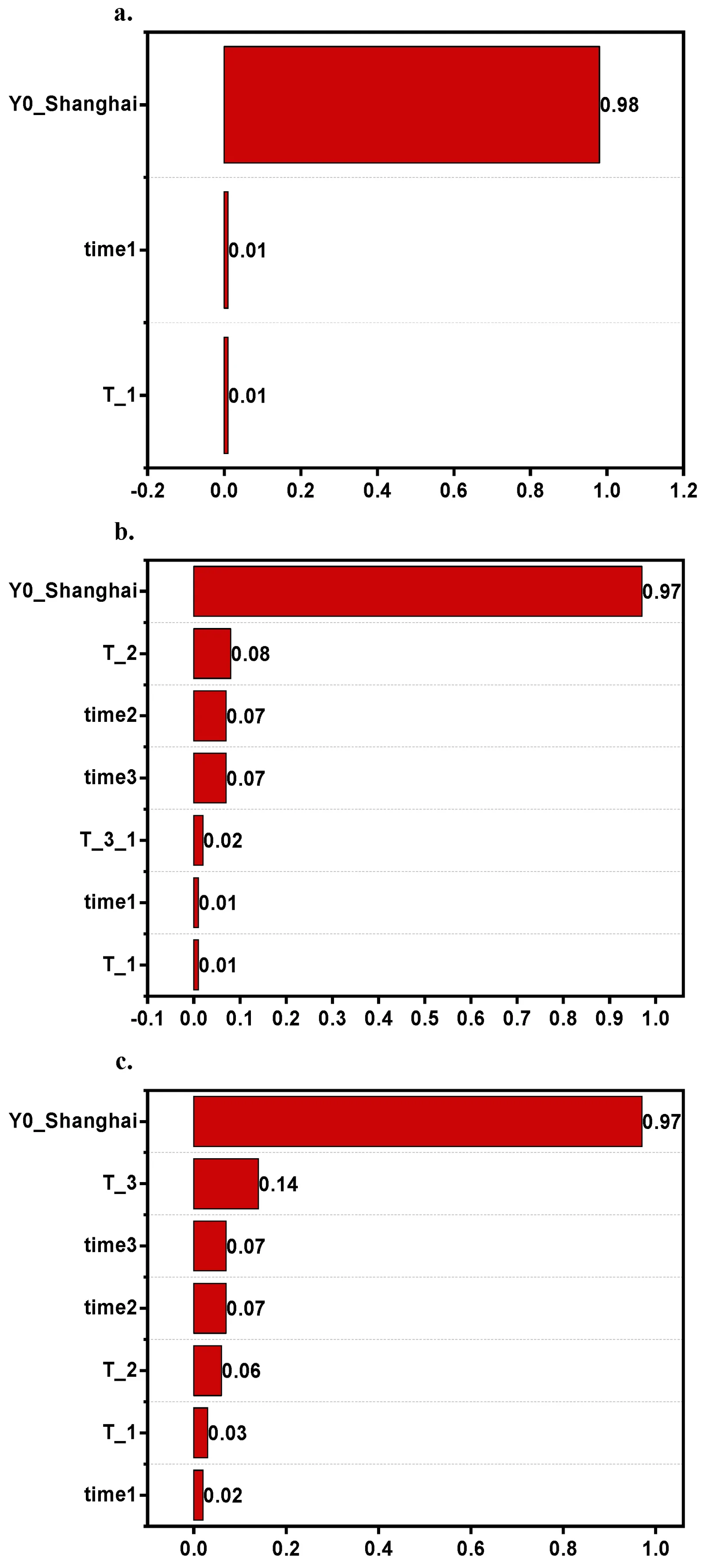
Sensitivity analysis of (**a**) Scenario 1, (**b**) Scenario 2, and (**c**) Scenario 3.

Figure 5 illustrates the sensitivity analysis results of three typical consumption scenarios, presented as Spearman rank correlation coefficients between the model input variables and the final risk estimates. The initial contamination level (Y_0__shanghai) consistently dominated risk across all scenarios, correlating most strongly with final risk estimates. As shown in Figure 5b, sensitivity analysis revealed that risk estimates in Scenario 2 were highly sensitive to transit conditions, including temperature (T_2) and duration (time2). In contrast, for Scenario 3, the domestic refrigeration temperature (T_3_2) exerted a significantly more pronounced impact on the final risk estimates than the parameters in the transit stage (Figure 5c).

## 4. Discussion

The pathogenic contamination of the RTE food results in severe foodborne illnesses, significantly threatening public health. Many international organizations, such as the Food and Agriculture Organization/World Health Organization (FAO/WHO, 2004) [24], the U.S. Food and Drug Administration/Food Safety and Inspection Service (FDA/FSIS, 2003) [49], and the European Food Safety Authority (EFSA BIOHAZ Panel, 2018) [50], have conducted comprehensive risk assessments to better characterize and manage the associated microbiological risks in RTE foods. These risk assessments provided a systematic framework for evaluating scientific evidence to estimate the probability of adverse health outcomes from contaminated RTE foods. However, risk assessments targeting regionally characteristic RTE products remain limited, primarily due to insufficient data availability. Campagnollo et al. [51] assessed the risk of listeriosis in traditional Minas Cheeses by compiling *Listeria monocytogenes* contamination data for different cheese types. The results showed that, for fresh soft cheese, the probability of listeriosis per serving in the general population was 3.44 × 10^−1^ ± 4.49 × 10^−1^, which was significantly higher than that of the artisanal semi-hard cheese (5.73 × 10^−5^ ± 5.3 × 10^−5^). These observations indicated that, even within the same food category, microbial risk can vary by several orders of magnitude, highlighting the necessity of product-level risk assessment.

Shanghai-style poached chicken is an RTE poultry product. In this limited survey, the estimated overall contamination rate of the three foodborne pathogens, including *Staphylococcus aureus*, *Listeria monocytogenes*, and *Salmonella,* was 8.33%, which showed a relatively higher observed prevalence compared with some RTE poultry products reported in previous studies. For example, Li et al. [52] conducted a contamination survey of salted duck in Nanjing, China, reporting an overall contamination rate of 3% for these three targeted pathogens. Notably, only *Staphylococcus aureus* was detected, while *Listeria monocytogenes* and *Salmonella* were not found in the salted ducks. Osaili et al. [53] investigated poultry products in Jordan and found that the contamination rates of *Salmonella* and *Listeria monocytogenes* were only 0.8% and 2.7%, respectively, in poultry products from Jordan. The relatively higher observed prevalence in this limited survey might be influenced by various aspects, such as product characteristics, sampling strategies, and microbiological detection approaches. For example, different from Shanghai-style poached chicken, salted duck is typically subjected to sufficient thermal processing followed by salting and seasoning procedures during production. These product characteristics may influence the survival and growth of microorganisms. In addition, salted duck samples were collected from local farmers’ markets over a full year, while the present study involved sampling from multiple retail settings within a relatively concentrated period. Variations in sampling locations and temporal coverage may also contribute to differences in the observed contamination levels of foodborne pathogens.

In addition, *Salmonella* exhibited the highest prevalence in Shanghai-style poached chicken among the three pathogens. Several studies have shown that *Salmonella* is one of the most prevalent foodborne pathogens in poultry and poultry products [54,55,56]. Therefore, in addition to cross-contamination, the traditional blanching-and-cooling process may result in non-uniform heating, allowing *Salmonella* to survive in some chicken carcasses. Based on semi-quantitative risk assessment, *Salmonella* showed the highest risk among the three pathogens, which was considered the primary microbial hazard requiring priority control during Shanghai-style poached chicken production and retail.

This study developed a growth model for *Salmonella* in poached chicken using isolated strains, which may improve the applicability and reliability of model predictions. The growth model fitting yielded an estimated theoretical minimum growth temperature parameter (T_min_) of 3.71 °C, which is slightly lower than reported literature ranges in media and meat products (typically 5–7 °C) [57,58]. The difference might be attributed to the rich protein and moisture content of poached chicken, as well as the reduction of competing microflora following thermal processing, which together might create more favorable conditions for the survival and growth of *Salmonella* at lower temperatures.

By integrating the *Salmonella* growth model with relevant consumption data, this study further assessed the probability of infection under three scenarios. Compared with dine-in consumption (Scenario 1), ambient storage (Scenario 2) and refrigeration (Scenario 3) increased the risk of illness by approximately 4-fold and 2-fold, respectively. These findings indicated that prolonged storage scenarios involving possible temperature variations may contribute to gradual risk accumulation. Sant’Ana et al. [59] reported a per-serving *Salmonella* infection risk of 5.7 × 10^−3^ ± 1.3 × 10^−2^ for RTE vegetables in Brazil, which was generally higher than the estimate in this study, likely due to differences in contamination sources between the two food categories. RTE vegetables are primarily subject to cross-contamination during harvesting, washing, and cutting, thereby often maintaining relatively high initial contamination levels. In contrast, although poached chicken undergoes thermal processing that reduces the initial microbial load, survival risk may persist due to non-uniform heating and the post-cooking handling process.

The sensitivity analysis indicated that the initial contamination level (Y_0__Shanghai) of *Salmonella* in poached chicken was the most influential risk factor. Bohaychuk et al. [60] reported a lower prevalence (3%) in thermally treated RTE chicken sausages in Alberta, Canada, compared with poached chicken. This might be attributed to the industrialized and standardized processing of RTE chicken sausages, which typically involves sufficient thermal treatment and vacuum packaging. However, further increasing thermal processing intensity to reduce the initial *Salmonella* load in poached chicken is not feasible, as it would compromise its traditional sensory and processing characteristics. Therefore, controlling *Salmonella* contamination at both the pre-heating stage (farm and slaughtering) and post-heating stage (retail handling) may be an important strategy for reducing Y_0__Shanghai.

Uncertainty is an inherent aspect of risk analysis, typically resulting from limited knowledge of parameters used in risk assessment models. In this study, certain parameters were derived from references or assumptions due to the lack of comprehensive data, which introduced uncertainty into the QMRA framework.

Firstly, the microbiological contamination data in this study were obtained from a limited number of samples (n = 84) collected during a single sampling period (July 2025). The relatively limited dataset and the restricted sampling period may not fully capture the variations in contamination levels across different conditions. Moreover, only four *Salmonella*-positive samples were detected, and only one sample had a quantifiable concentration, while the other three positive samples were below the LOQ. Therefore, the obtained contamination distribution used in the QMRA was partly based on the assumptions for sub-LOQ samples, which might have increased the uncertainty of the risk estimates.

Second, due to the lack of specific consumption data for poached chicken, RTE meat data were used as surrogates, potentially introducing uncertainty into exposure estimates. In addition, model parameters describing storage durations and temperature profiles were derived from published food-handling studies, local observations, expert knowledge, and summer meteorological records. PERT distributions were applied to mitigate extreme-boundary bias by prioritizing the most likely values. However, these parameters may not fully represent actual consumer handling conditions, introducing additional uncertainty into the quantitative risk assessment framework. Regarding the predictive growth model, although the Baranyi model and the Huang square-root model showed good fits to the experimental data, model validation was limited due to the small number of observations and the lack of independent datasets. Furthermore, in Scenario 3 of the exposure assessment, microbial growth under refrigerated conditions was predicted using the model beyond the experimentally validated temperature range (below 15 °C), which involved temperature extrapolation. The limited model validation and extrapolation beyond the tested temperature range may introduce additional uncertainty into the QMRA estimates.

Third, the dose-response model used in this study was derived from published international studies rather than local Chinese populations or *Salmonella* strains. Differences in strain characteristics, host susceptibility, and dietary patterns may introduce uncertainty into the predicted infection probability. Therefore, the quantitative risk estimates obtained in this study should be interpreted as comparative risk indicators among different exposure scenarios rather than precise predictions of absolute disease incidence. Future studies incorporating larger sample sizes, multi-season surveillance, and more comprehensive quantitative contamination data are needed to further improve the robustness of QMRA predictions. Moreover, the present study applied a one-dimensional Monte Carlo simulation, in which variability in exposure parameters was considered, while parameter uncertainty was not separately quantified. This may contribute additional uncertainty to the estimated risk results.

## 5. Conclusions

In conclusion, this study characterized the prevalence of three major foodborne pathogens in poached chicken retailed in Shanghai, including *Salmonella*, *Staphylococcus aureus*, and *Listeria monocytogenes*. Semi-quantitative risk assessment identified *Salmonella* as the most significant hazard. Integrating growth models of isolated strains, the estimated risk followed the order: room-temperature storage (Scenario 2) > refrigerated storage (Scenario 3) > dine-in consumption (Scenario 1). Sensitivity analysis highlighted the initial contamination level as the most influential parameter. Appropriate environmental hygiene practices to reduce the pathogenic contamination are considered as an important risk-management strategy. Moreover, the limited sample size, single sampling period, censored microbiological data, and extrapolation of predictive growth models beyond experimentally validated temperature ranges introduce inherent uncertainties into the QMRA predictions. Nevertheless, considering these findings alongside traditional preparation practices, enhancing environmental hygiene is identified as the key risk-management implication. This approach effectively mitigates public health risks while safeguarding the sensory and cultural identity of Shanghai-style poached chicken.

## Figures and Tables

**Table 2:** Semi-quantitative risk ranking results.

Outputs	*S. aureus*	*L. monocytogenes*	*Salmonella*
Predicted daily morbidity rate per person	1.74 × 10^−7^	1.08 × 10^−8^	4.17 × 10^−7^
Predicted cases per annum	1180	71	2830
Risk Score	44	42	52
Risk level	Medium risk	Medium risk	High risk

**Table 3:** Maximum specific growth rates and lag time estimated by Baranyi model.

Temperature (°C)	*μ*_max_ (h^−1^)	λ (h)
15	0.24	7.88
20	0.35	4.28
25	0.77	2.53
30	0.84	1.03
35	1.09	0.73
40	1.43	0.18

**Table 4:** Risk of *Salmonella* infection under three scenarios.

	Scenario 1	Scenario 2	Scenario 3
Different scenarios	Dine-in	Short-term ambient storage	Long-term refrigerated storage
Probability of infection (*Pinf*)	5.320 × 10^−6^	2.234 × 10^−5^	1.168 × 10^−5^

## Data Availability

The original contributions presented in this study are included in the article/Supplementary Materials. Further inquiries can be directed to the corresponding author.

## Supplementary Materials

The following supporting information can be downloaded at: https://www.mdpi.com/article/10.3390/foods15162908/s1, Table S1. Sampling locations and sample distribution; Table S2. Prevalence of *S. aureus*, *L. monocytogenes* and *Salmonella* in Poached chicken samples; Table S3. Concentrations of pathogens in positive samples.; Table S4. Variable parameter table for semi-quantitative risk assessment of poached chicken from retail to the consumption stage [61]; Table S5. Goodness of fit statistics for primary growth models of *Salmonella* in Poached chicken; Table S6. Estimated maximum population density (*Y*_max_, log10 CFU/g; *Y*_max_ = lg *N*_max_), standard error (SE), and 95% confidence intervals for Baranyi and Gompertz growth models under different incubation temperatures.
