# Supplementary material for: Risk Assessment of Foodborne Pathogens in Ready-to-Eat Poached Chicken in Shanghai, China
Source: Foods. 2026 Aug 19;15(16):2908. doi: 10.3390/foods15162908 (PMC13512114; doi:10.3390/foods15162908)
Supplement: Supplementary file 1 [file foods-15-02908-s001.zip › foods-4465513-supplementary.pdf]

Supporting Information

Risk Assessment of Foodborne Pathogens in Ready-to-Eat Poached Chicken in Shanghai, China

Qiuping Tian [1,†], Hua Cai [2,†], Hao Zheng [1], Xiang Wang [1], Zhuosi Li [1], Yangtai Liu [1] and Yue Ma [1,*]

[1] School of Health Science and Engineering, University of Shanghai for Science and Technology, Shanghai 200093, China; 243452636@st.usst.edu.cn (Q.T.); 243452611@st.usst.edu.cn (H.Z.); xiang.wang@usst.edu.cn (X.W.); lizhuosi@usst.edu.cn (Z.L.); usstlyt@163.com (Y.L.)
[2] Shanghai Municipal Center for Disease Control and Prevention, Shanghai 200336, China; caihua@scdc.sh.cn
* Correspondence: yuema@usst.edu.cn
† These authors contributed equally to this work.

Total Tables: **6**

Total number of pages: **5**

Table S1. Sampling locations and sample distribution.

District	Cooked Food Counters (Takeaway only)	Ordinary Restaurants (Takeaway & Dine-in)	Star-rated Restaurants (Dine-in only)	Total
Pudong	15	10	1	26
Minhang	7	5	3	14
Songjiang	4	2	1	7
Baoshan	3	4	0	7
Jiading	3	4	0	7
Yangpu	4	1	0	5
Xuhui	2	2	0	4
Hongkou	2	0	1	3
Changning	3	0	0	3
Jingan	2	1	0	3
Qingpu	3	0	0	3
Huangpu	1	1	0	2
Putuo	1	1	0	2
Total	47	31	6	84

Table S2. Prevalence of *S. aureus*, *L. monocytogenes* and *Salmonella* in Poached chicken samples.

Sampling	*S. aureus*		*L. monocytogenes*		*Salmonella*		Total
	Positive Sample	Prevalence %	Number of Detected Copies	Detection Rate %	Number of Detected Copies	Detection Rate %	Detection Rate %
Total (n=84)	2	2.38	1	1.19	4	4.76	8.33

Table S3. Concentrations of pathogens in positive samples.

	S. aureus	*L. monocytogenes*	*Salmonella*
positive sample 1 (Lg CFU/g)	4.60	3.26	1
positive sample 2 (Lg CFU/g)	5.78	-	1
positive sample 3 (Lg CFU/g)	-	-	1
positive sample 4 (Lg CFU/g)	-	-	4.53

Table S4. Variable parameter table for semi-quantitative risk assessment of poached chicken from retail to the consumption stage.

	Risk Ranger Input Parameters	*Staphylococcus aureus*	*Listeria monocytogenes*	*Salmonella*	Source/Rationale & Reference
Q1	Severity of hazard	Mild hazard	Moderate hazard	Moderate hazard	Li et al. [52]
Q2	Susceptibility of the relevant population	General population	General population	General population	Li et al. [52]
Q3	Consumption frequency (days consuming 100 g per time)	4.11	4.11	4.11	Calculated
Q4	Proportion of consuming population (%)	75	75	75	Wang et al. [48]
Q5	Number of consuming population	24,802,600	24,802,600	24,802,600	Population Census [47]
Q6	Prevalence rate (%)	2.38	1.19	4.76	Surveillance data from this study
Q7	Impact of post-processing operations	No impact	No impact	No impact	Product characteristic
Q8	Recontamination rate during post-processing operations	1%	1%	1%	Field investigation
Q9	Control the level of post-processing operations	Controlled (mostly reliable system in place results in 3-fold increase)	Controlled (mostly reliable system in place results in 3-fold increase)	Controlled (mostly reliable system in place results in 3-fold increase)	Li et al. [52]
Q10	Infectious dose increment for illness onset[S3]	10^5	8.33×10^5	8.33×10^4	FDA Bad Bug Book [20]; Kothary & Babu [14]; Mürmann, L. et al. [61]
Q11	Impact of pre-consumption treatment	No impact	No impact	No impact	Without home reheating.

Table S5. Goodness-of-fit statistics for primary growth models of *Salmonella* in Poached chicken.

Temperature/℃	MSE		RMSE		AIC	
	Baranyi Model	Gompertz Model	Baranyi Model	Gompertz Model	Baranyi Model	Gompertz Model
15	0.0288	0.0401	0.1697	0.2001	0.9067	4.2034
20	0.026	0.0242	0.1612	0.1556	-0.1201	-0.8289
25	0.0497	0.0669	0.223	0.2587	6.5325	9.2017
30	0.0223	0.0561	0.1493	0.2368	1.2365	7.6983
35	0.0272	0.0398	0.1648	0.1994	1.856	4.9064
40	0.0049	0.0055	0.0701	0.0738	-9.3504	-8.6158

Table S6. Estimated maximum population density (Y_{max}, log10 CFU/g; $Y_{max} = \lg N_{max}$), standard error (SE), and 95% confidence intervals for Baranyi and Gompertz growth models under different incubation temperatures.

Temperature/℃	*Y_{max} (log_{10} CFU/g)*		*Standard Error (SE)*		*95% CI Lower*		*95% CI Upper*	
	**Baranyi Model**	**Gompertz Model**	**Baranyi Model**	**Gompertz Model**	**Baranyi Model**	**Gompertz Model**	**Baranyi Model**	**Gompertz Model**
15	8.37	8.53	0.21	0.31	7.87	7.78	8.87	9.28
20	10.27	10.6	0.17	0.23	9.86	10.03	10.69	11.16
25	9.84	10.02	0.17	0.23	9.39	9.42	10.29	10.61
30	9.59	9.62	0.18	0.2	9.01	8.98	10.16	10.27
35	9.96	10.17	0.17	0.28	9.48	9.39	10.44	10.95
40	9.65	9.72	0.08	0.09	9.41	9.44	9.89	9.99
